# The utility of a modified WHO TB screening tool among children at a Botswana child welfare clinic

**DOI:** 10.4314/ahs.v21i1.11S

**Published:** 2021-05

**Authors:** Wame Dikobe, Mooketsi Molefi, Bornapate Nkomo, Botshelo Kgwaadira, Boingotlo Gasenelwe, Esther Seloilwe, Yohanna Mashalla, Tonya-Ascortt Mills

**Affiliations:** 1 Ministry of Health, Department of Public Health, TB Program, Gaborone, Botswana; 2 University of Botswana, Department of Family Medicine & Public Health, Gaborone, Botswana; 3 Botswana-University of Pennsylvania Partnership, TB Program, Gaborone, Botswana; 4 World Health Organization, Country Office, Gaborone, Botswana; 5 University of Botswana, Faculty Health Sciences, Gaborone, Botswana

**Keywords:** Childhood TB screening, HIV Exposure screening, TB/HIV integration

## Abstract

**Background:**

In high TB/HIV settings, the increased risk for TB amongst children exposed to HIV has been established through biomedical tests. Screening HIV exposed children for TB can improve early childhood TB detection and treatment.

**Objective:**

This study assessed the utility of a modified World Health Organization (WHO) tool by including HIV variables, to determine TB exposure amongst HIV exposed children presenting to a “Well Child” Clinic (CWC).

**Methods:**

Clinical data were obtained from medical records and/or from the caregivers of children presenting to CWC. Data was analyzed to explore factors associated with positive screening for TB, including being exposed to HIV and current HIV status.

**Results:**

Five percent (55/1100) screened reported a close TB contact and 21% (n=231) had positive TB symptom screen. History of close TB contact was a risk factor for positive screening for TB symptoms (OR 1.89 CI 1.05–3.4) while being HIV negative was protective (OR 0.3, Cl 0.19–0.62). HIV exposure was associated with increased risk of TB exposure (OR 2.9 CI 1.61–5.19).

**Conclusion:**

Integrating HIV variables in the existing WHO screening tool for childhood TB can be useful in early detection and treatment of TB in HIV exposed children in resource limited settings.

## Introduction

Childhood Tuberculosis (TB) is considered a silent epidemic since cases are often missed or underreported due to their unique presentations with atypical symptoms leading to diagnostic difficulties and the challenges of bacteriological confirmation.^1^–^5^ Globally in 2017, the World Health Organization (WHO) estimated 1 million new childhood TB cases, out of the reported 10 million TB cases.^1^ In sub-Saharan Africa, the burden of TB is further fueled by Human Immunodeficiency Virus (HIV), with 70% of all people living with HIV/TB co-infection worldwide.^1^ Though there has been a decline in TB cases, due to increased access to antiretroviral therapy (ART), Botswana still remains one of the countries with the highest estimated TB/HIV incidence rate.^1^ Over the past decades TB/HIV co-infection rate reduction from 86% in 2003 to 49% in 2018 was mirrored by a decline in TB notifications rates from 442/100,000 in 2008 to a rate of 275/100,000 in 2018.^1^,^6^,^7^

During the same period, PMTCT program was scaled-up and the country saw a decline in mother to child transmission rate from 42% in 2008 to 2.54% in 2018.^8^–^10^ Even though Botswana is considered a high burden TB country, case reporting of childhood TB cases remains relatively low, with only 6% of annual TB notifications being children aged 0–14 years.^1^ This discrepancy between the high TB rates and low childhood TB cases, suggests that there may be underreporting of the burden of childhood TB cases nationally. Community studies in the region, have shown a strong association between household exposure to an adult TB case and infection amongst young children.^11^–^15^ This risk is even higher in HIV exposed but HIV uninfected children (HEU) and HIV infected children who may be in close contact with a sputum negative or sputum positive caregiver. 16–18 HEU children have a higher risk for TB infection compared to children born to HIV uninfected mothers due to increased exposure to active TB and immunological factors affecting BCG vaccine response and increasing susceptibility to TB infection.^18^

Despite this clear association between HIV exposure and the risk for TB, there are no current recommendations to routinely screen this vulnerable subgroup for TB exposure, as proposed for HIV positive children^2^,^3^ resulting in a missed opportunity to place these children on TB prevention therapy (TPT). Additionally, over the years countries have done well in reducing mother to child transmission (MTCT) of HIV through rapid expansion of ART. Currently, Botswana has one of the lowest mother to child transmission rate(MTCT) of rates of 1.4%.^19^ However, there are gaps in post-natal follow-up of HIV exposed babies, with large proportion of children, up to 40% who are seen at local health facilitates with an “unknown” HIV status.^12^,^20^ Systematic reviews and meta-analysis of PMTCT in sub-Saharan Africa have shown similar results, with only 55% of exposed babies having early infant diagnostic testing at 12–18 months.^21^ Determining the HIV exposure of the child while screening for TB has the potential to close the loopholes that exist in the current PMTCT programs by identifying children who are often exposed and untested or tested but without any results. Integrating TB/HIV/Child Welfare services, in child wellness clinics can bridge the existing gaps in TB/HIV management by providing linkages to isoniazid preventative therapy (IPT) and offer HIV testing to HIV-exposed under-fives without documentation of HIV testing. Additionally, this strategy can be easily adapted by resource limited countries like Botswana, which have well-established and robust systems for routine immunization and weight monitoring of children under the age of five years. Children present to the “well clinic” (CWC) on a monthly basis for weighing and immunizations. Using this avenue to screen children for TB and HIV is a potentially cost-effective way of identifying vulnerable HIV-exposed children and tailoring interventions to meet their needs. This will result in early case- finding, leading to early initiation on either TPT or TB treatment for active TB cases, while providing an opportunity to strengthen the follow-up of children within the PMTCT cascade, a gap that persists in HIV programs.^8^–^10^

## Methods

### Study design and population

A cross-sectional descriptive study was conducted prospectively to assess the utility of a modified World Health Organization (WHO) by including HIV variables, to determine the risk of TB exposure amongst HIV exposed children presenting to a “Well Child” Clinic (CWC).The existing WHO screening tool was modified to include questions on HIV exposure in children presenting with a negative or unknown HIV status. The study was conducted in an urban clinic in Greater Gaborone, with a high burden of TB and HIV, between March 8^th^ and April 29^th^, 2016. All children under-five presenting to the CWC were screened for TB and screened for history of exposure to HIV using a modified WHO screening tool. Data was collected by Health Care Auxiliaries by asking the caregiver and reviewing the child's under-five cards. TB Positive screening was defined as per WHO TB symptom screen, as presence of any of the following symptoms; cough of any duration, fever, fatigue, failure to thrive and enlarged lymph nodes. Those who screened positive were referred to a clinician (nurse or doctor) for a full-assessment and evaluation in accordance with the National guidelines. HIV exposure was derived from the prevention of mother to child transmission (PMTCT) page in the under-five cards, and if undocumented recorded as such in the modified screening tool. Referrals were also made for further testing as per the national guidelines.

### Data collection

**Figure 1:**
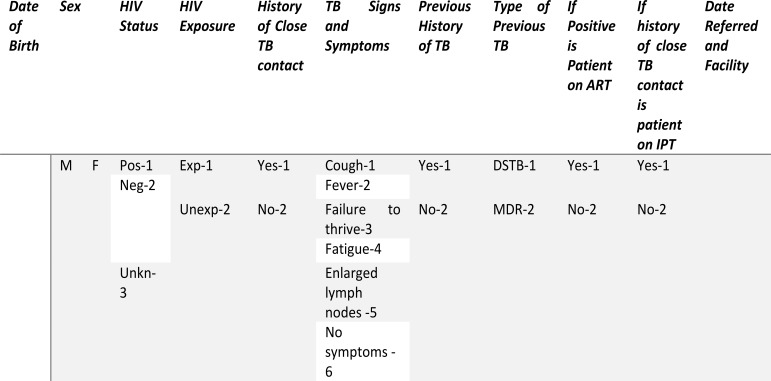
Modified WHO screening tool, integrating childhood TB/HIV variables

The WHO TB screening tool was modified for child specific TB symptoms to capture information on HIV exposure history of the child and implemented in Child Wellness Clinics (CWC) (figure 1). The tool was applied to all children for assessing both TB symptoms and exposure, and HIV exposure and status by the health care workers who were weighing the children.

Caregivers were asked to answer the standard TB symptom screen questions: (1) Is your child coughing? (2) Does your child have fever? (3) Does your child have fatigue or reduced playfulness? (4) Does your child have any enlarged lymph nodes? Failure to thrive and weight-loss were assessed objectively using the growth monitoring chart in the under-five clinic cards. Additional questions included history of close TB contact and history of taking TB treatment or IPT. Children who screened positive on any of the above questions, regardless of duration were referred to a nurse who determined what if any further evaluation was needed.

HIV status was determined using information from the under-five cards and if information was missing, it was elicited from the caregiver. Children who did not present with a guardian/parent with missing information were captured as “undocumented”. Contact information of the parent was taken, and phone follow-up made by the health auxiliary or the health education assistant.

Those who were HIV exposed but without a documented HIV test result as per the PMTCT guidelines, were referred for appropriate HIV testing services (early infant diagnostic (EID) services or HIV testing services) regardless of their TB screen results.

Data was first captured on a paper-based registry before transfer to Epi-Info (CDC software). In addition to the variables described above, demographic data on age and sex were also recorded. In order to minimize errors, data was entered by two different people, one reading and another entering, and a 3^rd^ person checked every 10th entry for quality assurance.

### Ethical Clearance

A waiver of consent was granted by the Botswana Ministry of Health Ethics Committee because this was part of routine care and in accordance with Botswana National Guidelines for TB and HIV. Privacy and confidentiality, voluntary participation and data anonymization at reporting were maintained in the study. Ethical clearance was granted by the Botswana Ministry of Health, Human Research Ethics Committee (HPDME 13/18/1 X (363).

### Statistical analysis

Analyses were conducted using STATA 14. Descriptive statistics were used to describe frequencies, such as gender, age distribution, and proportion with positive symptoms, proportion with close TB contact and HIV status and exposure. Chi-square test was performed to test for significance of association between demographic and TB exposure variables (age, sex, HIV status, HIV exposure, TB symptoms). Logistic regression was used to assess correlation of variables and variables that are significantly associated with the TB positive screen were identified by computing the 95% confidence intervals and p-value less than 0.05 to be considered statistically significant.

## Results

**Figure 2:**
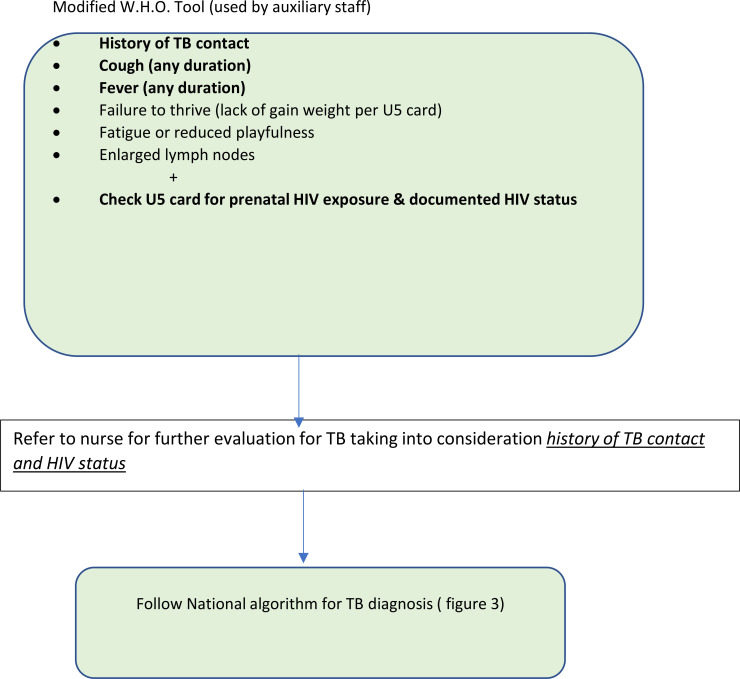
Flow-chart during study implementation of the modified W.H.O. tool

In two months, a total of 1100 children were screened at the intervention site, comprising of 80% of all children who presented the health facility during the study period (Figure 2). Of these 280 were excluded due to missing unique identifier or duplicate screening.

### TB symptom screen

**Figure 3:**
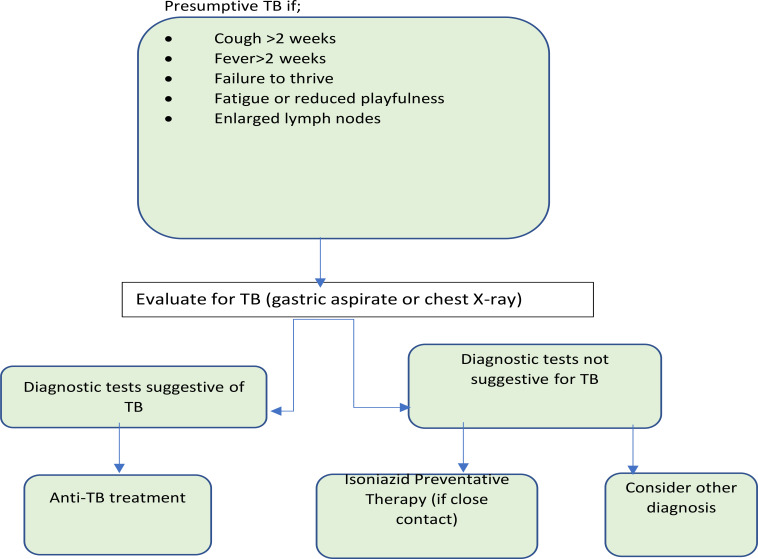
Algorithm for diagnosis of TB in children <12 years old

Of the 1,100 children screened 52.4% (n=582) were male and 75.5% (n=838) were under 3 years Table 2). Twenty-one percent (n=231) had positive TB symptom screen, with 75.3% (n=174) reporting cough as the most common presenting symptom, 50% reporting cough alone (n=116) and the rest reporting cough in association with other symptoms (Figure 3). Fever was the single most common associated symptom with cough at 21.6% (n=50). About 14% (n=32) presented with failure to thrive (FTT) alone and 18% (n=42) with FFT associated with other symptoms. Of the 21% (n=231) who had a positive symptom, only 43.3% (n=100) were referred to the nurse for further evaluation by the health auxiliary.

**Table 2:** Factors associated with positive TB symptom screening

Characteristics		Frequency n (%)	OR	95% CI	OR	95% CI
			unadjusted		Adjusted*	
Sex	Female	528 (47.6%)	0.77	0.58–1-04		
	Male	582 (52.4%)	1			
Age	0–3yrs	838 (75.5%)	0.83	0.58–1.18		
	4–5yrs	272 (24.7%)	1			
HIV Status	HIV Positive	14 (1.3%)	1			
	HIV Negative	214 (19.2%)	0.25	0.8–0.74	0.3	0.19–0.62
	HIV unknown	882 (79.4%)				
HIV exposure	HIV exposed	255 (22.9%)	1.09	0.55–2.18		
	HIV unexposed	798 (71.9%)	1			
	Undocumented status	57 (5.1%)				
History of TB contact	Yes	55 (5%)	2.02	1.14–3.57	1.89	1.05–3.4
	No	1055 (95%)	1			

* Adjusting for HIV exposure

### HIV status of HIV exposed children

**Figure 4:**
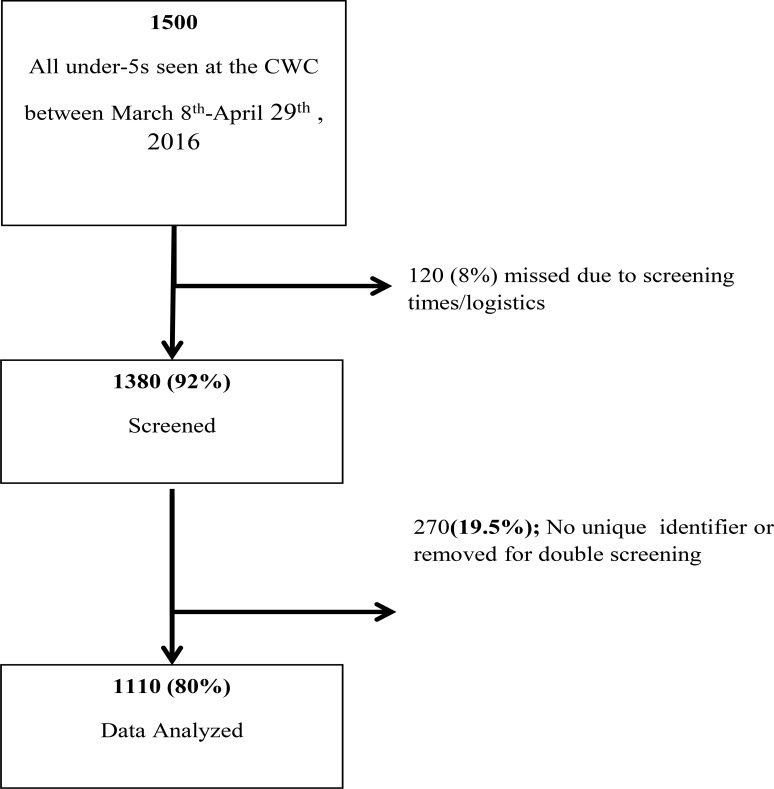
Flow-diagram of children presenting to CWC

History of exposure to HIV was recorded from the PMTCT section of the under-five cards and took into account both child and the maternal HIV status during the prenatal period. About 1.3% (n=14) of all had a positive HIV test documented, while 85% (214/255) of those who were documented as exposed had a negative HIV test recorded (Figure 4). The majority (87%, 768/882) of children who did not have an HIV status (unknown status) recorded were documented as HIV unexposed during the prenatal period, based on the maternal antenatal HIV test result in the PMTCT records. However, about 7% (59/882) of those who had an unknown status had been born to HIV positive mothers. All (59) children were referred to the nurse for further evaluation and referral.

### HIV Exposure vs TB Exposure

Fifty-five (5%) of children reported a close TB contact. Of the children who reported a history of close TB contact, 33% (n=18) and 45% (n=26) were symptomatic and HIV exposed respectively. Being exposed to HIV was statistically associated with increased risk of TB exposure (OR 2.9, CI 1.61–5.19, p=0.0001).

Logistic regression was used to determine association between demographics, HIV exposure, HIV status and TB contact with a positive TB symptom screen (Table 3). History of a close TB contact was associated with screening positive for TB symptoms (OR 1.89, Cl 1.05–3.4). Additionally, being HIV negative was associated with being less likely to screen for TB symptoms (OR 0.3, Cl 0.19–0.62).

## Discussion

This cross-sectional survey revealed that there is a 5% prevalence of TB exposure (exposure to a positive TB household contact) in the children presenting to this urban CWC in a high burden TB/HIV are in Gaborone, during the study period. The prevalence of TB exposure in the national under-five population is unknown as this data is not aggregated and reported to the Botswana National TB program. Thus, we cannot compare the 5% found at this one site to the national or other areas in the country. However, it does suggest that TB exposure in high burden areas is significant.

Previous studies in hospital setting, have shown a strong correlation between TB contact and confirmed TB in high-risk children who were non-HIV-infected and aged <3 years.^12^ Furthermore, children who are HIV exposed, even if HIV uninfected, are born to HIV infected mothers, who are at a higher risk of TB in high TB/HIV settings.^16^–^18^ Our study found HIV exposure to be statistically associated with TB exposure, consistent with studies that have suggested this association. A study in rural Uganda, found that HIV exposed uninfected (HEU) children had significantly higher odds of TB infection by QuantiFERON (OR 21.2, p 0.008, 95% Cl 2.2–204.7).17 This study was able to illustrate these association via a screening questionnaire without any diagnostic test, such as TST or QuantiFERON (QFT), suggesting that there may be yield in incorporating HIV exposure and testing variables in the standard TB screening tools. This further supports that HIV exposed children are vulnerable to TB, because they are more likely to have confirmed TB caregivers, therefore screening HIV exposed children in routine care maybe a higher yield for identifying active TB cases and determining TPT eligibility. The current recommendation is for active contact tracing for all household contacts of a smear positive TB case; however, this is seldom done due to resource limitations, missing TB disease in children. 16,18

Applying an integrated TB/HIV tool, that asks about HIV exposure to children presenting for routine CWC visits is a cost-effective, easily adaptable modality that will help to quickly assess for the risk of TB exposure in this vulnerable population.

Screening positive for TB symptoms was associated a history of a close TB contact independent of HIV exposure. A study in South Africa found that children aged 5–9 years diagnosed with positive TST were twice likely to have an adult TB contact, regardless of HIV status.13 On the contrary, we found that children with a negative HIV test were less likely to present with TB symptom, when compared to their HIV positive counterparts. This correlation is well-established since HIV positive children are more vulnerable to TB.^5^

Due to the limitation of the study, we were unable to report how many of the children with a contact or were symptomatic were evaluated for TB and eventually diagnosed with active TB versus needing TPT. Evaluating children for TB is not simple and often involves multiple diagnostic tests and referral to higher centers of care.^4^,^12^,^20^ A previous study in Botswana looking at children with failure thrive identified in a CWC clinic had a yield of 6% of those completing an evaluation needed TB treatment but there were high lost to follow up between identification of failure to thrive and a complete diagnostic evaluation.^20^ Thus, further studies are needed to determine of those with a positive screen by the tool utilized in this study how many actually have TB or need TPT and if more of these children come from the HIV exposed cohort.

### Gaps in PMTCT Cascade

Of the children screened, 882 (79.5%) had an unknown HIV status. However, when prenatal HIV exposure was determined, n=255 (22.9%) of the 1110, were exposed, and n=798 (71.9%) were unexposed, the prevalence of HIV in women of reproductive age (15–49) in 2016.10 The current policy is to only follow-up and test children who are known to be exposed unless they have symptoms or other risk factors for HIV (such as being hospitalized), which explains why most children with a known HIV test (either positive or negative), were those documented as HIV-exposed. However, interrogating the patient's HIV status, followed by the HIV exposure status, proved to be helpful in identifying HIV exposed babies who had not been tested, and referring them appropriately. During the study 69 HIV exposed, lost to follow-ups in the PMTCT cascade were referred for HIV testing services. This suggests that CWC clinics are good places to screen for infants lost to follow-up in PMTCT and ensure they are tested according to national guidelines.

## Limitations

The study is limited by the sample size and site selection, and therefore results may not be generalized to the entire population. However, it provides a glimpse in the magnitude of the problem.

## Conclusions

Integration of TB and HIV indicators into routine CWC activities was possible and proved beneficial in allowing for referral for HIV testing in children lost to follow up in PMTCT. In a high TB/HIV setting, 5% of children presenting to a well child clinic reported a positive TB contact at home, within the past 2 years, suggesting that routine screening of children for TB contacts in well child clinics may be fruitful to catch missed opportunities for TPT or undetected cases of active TB. In addition, because of the association between HIV exposure and TB exposure this study suggests that this group of young children may have a higher yield in TB screening and thus warrant increased focus. The actual yield of the screening for children started on TPT or TB treatment still needs further evaluation.

## Figures and Tables

**Table 1:** Definition of study terminology

Term	Definition
Close TB contact	A person with TB, living in the same compound or household with the child in the past 6 months
HIV exposed	A child born to a HIV positive mother, as recorded in the under-5 card
HIV unexposed	A child born to an HIV negative mother, as recorded in the under-5 card
Positive TB screen	Screening positive for TB symptoms as per WHO guidelines, as having any of the following symptoms; cough of any duration, fever, fatigue, failure to thrive or enlarged lymph nodes
TB exposure	Having a close household TB contact
